# Occurrence, temporal trends, and half-lives of perfluoroalkyl acids (PFAAs) in occupational workers in China

**DOI:** 10.1038/srep38039

**Published:** 2016-12-01

**Authors:** Jianjie Fu, Yan Gao, Lin Cui, Thanh Wang, Yong Liang, Guangbo Qu, Bo Yuan, Yawei Wang, Aiqian Zhang, Guibin Jiang

**Affiliations:** 1 State Key Laboratory of Environmental Chemistry and Ecotoxicology, Research Center for Eco-Environmental Sciences, Chinese Academy of Sciences, Beijing 100085, China; 2 Institute of Environment and health, Jianghan University, Wuhan 430056, China; 3 School of Medicine, Jianghan University, Wuhan 430056, China

## Abstract

Paired serum and urine samples were collected from workers in a fluorochemical plant from 2008 to 2012 (n = 302) to investigate the level, temporal trends, and half-lives of PFAAs in workers of a fluorochemical plant. High levels of perfluorohexane sulfonate (PFHxS), perfluorooctanoic acid (PFOA), and perfluorooctanesulfonate (PFOS) were detected in serum with median concentrations of 764, 427, and 1725 ng mL^−1^, respectively. The half-lives of PFAAs in workers were estimated by daily clearance rates and annual decline rates of PFAAs in serum by a first-order model. The geometric mean and median value for PFHxS, PFOA, and PFOS were 14.7 and 11.7, 4.1 and 4.0, 32.6 and 21.6 years, respectively, by the daily clearance rates, and they were 3.6, 1.7, and 1.9 years estimated by annual decline rates. The half-lives estimated by the limited clearance route information could be considered as the upper limits for PFAAs, however, the huge difference between two estimated approaches indicated that there were other important elimination pathways of PFAAs other than renal clearance in human. The half-lives estimated by annual decline rates in the present study were the shortest values ever reported, and the intrinsic half-lives might even shorter due to the high levels of ongoing exposure to PFAAs.

Perfluoroalkyl acids (PFAAs) are a group of highly stable man-made compounds that are used in surfactants, fluorinated polymers, and fire-resistant foams because of their unique thermal and acid resistance, as well as their hydro- and lipophobic properties^1^. From 1970 to 2002, approximately 96,000 tonnes of perfluorooctane sulfonyl fluoride (POSF), a perfluorooctanesulfonate (PFOS) precursor, were produced, and approximately 450–2700 tonnes of PFOS entered wastewater streams^2^. The 3 M Company was the main PFOS producer in history and manufactured approximately 3600 tonnes per year of POSF before 2002^2^. PFAAs contamination was not only found around point sources, such as fluorochemical facilities^3^, but also detected in remote areas, such as the Tibetan Plateau^4^ and the Arctic^5^,^6^, due to their high historic production and continuous release. The adverse effects of PFAAs on humans, such as osteoarthritis and a delay of puberty, have been proposed^7^,^8^. They were also suspected to affect adult thyroid hormone function and increase certain carcinogenic activities^9^,^10^. The 3 M Company phased out PFOS production in 2002 due to its environmental persistence and potential toxicity. In 2009, the 4th meeting of the Conference of the Parties to the Stockholm Convention added PFOS, its salts and POSF into Annex B to restrict the production and usage of PFOS-related compounds, with exemptions for specific uses^11^. Consequently, decreasing trends in concentrations of PFOS in both environmental matrices and human body were observed^12^,^13^,^14^. Nevertheless, the production volume of PFAAs, including PFOS, has increased in China since then^15^, and PFAAs with shorter carbon chains, such as perfluorohexane sulfonate (PFHxS), have been produced as one of the major substitutes for PFOS. Our previous study evaluated the influence of a fluorochemical manufacturing facility on the ambient environment and found three major PFAAs (PFHxS, perfluorooctanoic acid (PFOA), and PFOS) at higher concentrations in the surrounding environment, indicating a high exposure risk for the workers^3^.

Moreover, PFAAs can bind to serum albumin and are prone to accumulate in the blood and liver of organisms^16^,^17^,^18^. As a result, PFAAs were frequently detected in various human serum samples. The serum concentration of PFAAs ranges from single-digit ng mL^−1^ levels in the general population to several μg mL^−1^ in occupationally exposed workers^19^,^20^,^21^,^22^,^23^,^24^,^25^,^26^. However, there are great differences among the elimination rates of the individual PFAAs in humans. The reported half-lives based on serum concentrations ranged from 26 days for PFBS to more than 5 years for PFHxS and PFOS^21^,^27^,^28^. Moreover, the data from different sources are even controversial for the same compound. Olsen *et al*. considered the half-life of PFOS for retired fluorochemical production workers to be approximately 5 years [95% CI, 3.9–6.9 years]^21^, while Zhang *et al*. estimated that the half-life of PFOS in males and older females exceeded 10 years (average ± SE: 27 ± 3.1 years)^28^. Half-lives of PFAAs in humans are directly associated with the elimination pathways, while the accumulation/elimination pathways in humans are still not well known. Renal elimination is one of the most critical processes in determining the total body clearance of perfluorocarboxylates (PFCAs), but other factors such as menstrual clearance and biliary excretion cannot be neglected^29^,^30^.

We collected paired serum and urine samples of occupational workers in a fluorochemical plant in China from 2008 to 2012 in this study, covering important time points before and after the PFOS restriction in 2009. The body burden and temporal trends of PFOA, PFOS, and PFHxS in the workers were then investigated. Furthermore, the half-lives of PFAAs in workers were estimated by both the PFAAs daily clearance and annual decline rates.

## Methods

### Sample Collection

The selected fluorochemical plant (Henxin Chemical Plant) in this study is one of the largest producers of PFOS-related chemicals in China and is located in Yingcheng, Hubei province^3^. Perfluoroalkyl sulfonic acids (PFSAs) and perfluorotertiary amines are synthesized through an electro-chemical fluorination (ECF) process. We collected paired serum and urine samples from the occupational workers in the plant for five consecutive years, and the sampling activities were carried out in Nov. 2008, Nov. 2009, Nov. 2010, Dec. 2011, and Dec 2012, respectively. The Medical Research Ethics Committee, School of Medicine, Jianghan University, approved the ethics of this study, and all methods were performed in accordance with the relevant guidelines and regulations, moreover, all volunteers gave written informed consent. Demographic information of the donors, including gender, age, length of service, and work assignment, was collected with a questionnaire (Table S1). The sampled population included a total of 302 occupational workers, of which there were 89 female and 213 male participants. In total, 302 serum and 274 urine samples were collected.

The participants were told not to eat breakfast before the sampling, and morning urine samples and blood samples were collected. Serum was separated from the blood by centrifugation at 1100 × g at 4 °C for 10 min and then placed on ice. Serum samples were then transferred to our laboratory on the same day and stored in polypropylene containers at −20 °C until analysis.

### Laboratory Analysis

All pre-treatment methods and instrument analysis of PFAAs in serum and urine samples are based on previous studies with minor modifications (see Supplementary Information)^3^,^31^. Based on the PFAA profiles in environmental matrices around the same plant in our previous study^3^, only 3 main PFAAs, PFHxS, PFOA, and PFOS, were analyzed in the obtained serum and urine samples. Instrumental analysis of the PFAAs was performed by a HPLC-ESI/MS/MS system (HPLC: Waters 2695, MS: Waters Quattro Premier XE). The limits of detection (LODs) of PFHxS, PFOA, and PFOS were 0.020, 0.063, and 0.018 ng mL^−1^ for serum samples and 0.008, 0.025, and 0.007 ng mL^−1^ for urine samples, respectively. A procedural blank was performed for every batch of seven samples, and all of the blank levels were below the LODs. The spiked recoveries of PFHxS, PFOA, and PFOS ranged from 95% to 110% for the serum samples and 111% to 123% for the urine samples, respectively. Details of quality assurance and quality control of the chemical analysis can be found in the Supplementary Information.

### Estimation of half-lives of PFAAs

Olsen *et al*.^21^ found linear relationship between the logarithm of serum PFAAs concentration and time, and a first order model was adopted to estimate the half-lives of PFAAs. The elimination of PFAAs in humans was also described with the following equation:


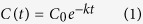


If we set C(t) as 1/2C_0_, then the half-life


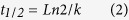


where *k* is the elimination rate constant. We calculated *k* using two approaches in the present study. In the first approach, *k* is defined as Cl_total_/V_d_, where V_d_ stands for the volume of distribution of PFAAs in the human body. Cl_total_ represents the total daily PFAAs clearance in the human body; for men and women older than 50, it refers to renal clearance, while for young women, it is defined as the sum of menstrual clearance and renal clearance. To keep consistency with other studies, V_d_ of PFOS and PFHxS were set at 230 mL kg^−1^, and V_d_ of PFOA was set at 170 mL kg^−1^ in this study^28^,^32^,^33^. In the second approach, *k* is defined as the average annual decline rates of PFAAs in workers who participated in this study.

### Worker Grouping and Statistical Analysis

The PFAA body burden might relate to uncontrolled factors such as the age and gender of the donors. Therefore, the donors were grouped by age, BMI (weight divided by the square of height (kg m^−2^)), gender, length of service, and work assignment in this plant. Based on the Chinese standard of BMI, the workers were divided into three sub-groups: underweight donors with BMI lower than 18, normal donors with BMI between 18 and 24, and overweight ones with BMI over 24^34^. For the length of service in the plant, 3 categories, less than 1 year, 1 to 3 years, and over 3 years, were considered. Considering the specific work assignments, there were five sub-groups: the workers in the sulfonation department, the fabric finishing agent department, the electrolytic process department, the research and development department, and the management office. Taking age and gender into consideration, young (≤50) and old (>50) and male or female were used as classification criteria. Different from the participants in the previous study, 82.3% of the workers in this study are under 50 years old. 78.5% of the workers had a BMI of 18 to 24, with only 18.9% overweight, although both the mean BMI value and the cut-off point for overweight for the Chinese are much lower than those of Western people and the existing WHO classification. A detailed statistical description of the factors such as the age, BMI, and gender of the groups is shown in Table S1 in the Supplementary Information. The PFAA concentrations were set to LOD/2 if the concentrations in the samples were below the LOD for further statistical analysis. Spearman correlation coefficients between levels were used for the correlation analysis among the levels of different PFAAs. The frequency distributions of the serum PFAAs suggested log-normal distributions, which were further confirmed by the Shapiro-Wilk test. Using the concentrations of PFAAs as continuous dependent variables, the models using log-transformed variates fit the data better than a model using untransformed variates. Moreover, a specific general linear model was obtained by an analysis of covariance, in which the potentially confounding covariates BMI and age were set as continuous variates, while the other factors were set as categorical variates. All statistical analyses were performed using SPSS statistical software (Version 17.0).

## Results

The serum concentrations of PFHxS, PFOA, and PFOS in workers were in the ranges of <LOD to 19,837 ng mL^−1^ (median = 764 ng mL^−1^), 2.52 to 32,000 ng mL^−1^ (median = 427 ng mL^−1^), and 50.3 to 118,000 ng mL^−1^(median = 1,725 ng mL^−1^), respectively (Table 1). The detection frequency of PFHxS, PFOA, and PFOS in the urine samples of the study were 90%, 93%, and 91%, respectively. The urine concentration of PFHxS, PFOA, and PFOS in the workers ranged from <LOD to 34.0 ng mL^−1^, <LOD to 53.6 ng mL^−1^, and <LOD to 81.5 ng mL^−1^, respectively, with the median values of 1.3, 2.1, and 1.3 ng mL^−1^, respectively. Serum concentrations of PFAAs in male workers were significantly higher than those in female workers when checked with a Mann-Whitney non-parametric test (*p* < 0.05; n male = 213, n female = 89) (Figure S1). The average concentrations of serum PFHxS, PFOA, and PFOS in males (Average ± SD) reached 2,191 ± 3,050, 1,215 ± 2,936, and 7,040 ± 15382 ng mL^−1^, while they were 1,044 ± 1718, 659 ± 743 and 2,221 ± 4,791 ng mL^−1^ in females, respectively. However, no significantly difference was found between female workers and male workers in individual departments. The higher PFAAs body burden in male workers might due to their high proportion in this plant, male workers accounted for 70%. Serum PFAA levels in occupational settings were much higher than those in the general population^19^,^23^,^26^. Serum PFOS concentrations in 85 percent of the sampled Chinese population were less than 100 ng mL^−1^ 25, whereas in the present study, the value, 6775 ng mL^−1^, was almost 68-fold higher. Concentrations of PFHxS and PFOA in serum of general population were ranged from the magnitude of 0.1 ng mL^−1^ to 10 ng mL^−1^ 22,23,25, however, geometric mean of serum PFHxS and PFOA reached 628 and 334 ng mL^−1^ in occupational workers, respectively, which were several orders of magnitude larger than those in general population. The serum PFOS and PFHxS concentrations of the workers in this plant were much higher than workers in another fluorochemical plant in China, in which the median serum PFOS and PFHxS were 33.5 and 0.98 ng mL^−1^, respectively^24^. Concentration of serum PFOS (geometric mean) reached 1677 ng mL^−1^ in our case, which also exceeded the contamination level in the workers of the 3 M Company, who exhibited serum PFOS (geometric mean) of 910 ng mL^−1^ 20. On the other hand, the serum PFOA here (334 ng mL^−1^) was lower than in previous occupational studies, and the geometric mean of serum PFOA in occupational workers of Olsen *et al*.’s^20^ and Wang *et al*.’s^24^ study was 1130 ng mL^−1^ and 1272 ng mL^−1^, respectively. The discrepant product structure of the relevant plants might be the cause of such differences. PFOA is a product or raw material for both the 3 M Company^20^ and the fluorochemical plant in Wang *et al*.’s study^24^, while the plant in our study only produces PFSAs or PFSA-based products, while PFOA is considered as a by-product.

PFAA concentrations in the sub-groups divided by work assignment are shown in Table 1. Serum PFOS and PFHxS were highest in workers of the sulfonation department, although the serum PFOA levels in the workers possessed unique characteristics, as the highest serum PFOA concentrations were detected in the workers from the electrolytic process department. As PFOA was not the product of this plant, it might be suggested that PFOA was primarily formed during the electro-chemical fluorination process. The rank orders of serum PFAA concentrations for the workers were exactly consistent with that for dust samples obtained in the corresponding departments^3^, and our previous study reported that dust ingestion was a very important exposure pathway in this plant^35^.

The serum levels of PFHxS and PFOS exhibited a similar profile in different departments, the concentration of serum PFHxS among the 5 departments followed the order of sulfonation department > fabric finishing agent department > electrolytic department > research building > management office, while the concentration of serum PFOS followed the order of sulfonation department > electrolytic department > research building > management office > fabric finishing agent department (Table 1). The fabric finishing agent department was established in 2010, with the workers here employed after PFOS was restricted, and PFHxS or PFHxS-based products are the raw material in this department. Therefore, high PFHxS exposure in this department was expected, but the workers there had a lesser and shorter exposure to PFOS. Significant correlations (*p* < 0.01) existed among the different PFAAs in all workers, the correlation coefficients for PFHxS and PFOA, PFHxS and PFOS, PFOA and PFOS were 0.76, 0.62, and 0.52, respectively. Moreover, the significant correlations (*p* < 0.05) among different PFAAs also found in workers form the same department (Table S2). These results indicated that these three compounds might have the same source and/or exposure pathway. The serum PFHxS, PFOA, and PFOS of the workers in the sulfonation, electrolytic process, and fabric finishing agent departments were much higher than those in the research and development department and management office. Obviously, direct contact with PFAAs results in high serum PFAAs in the workers.

**Figure 1:**
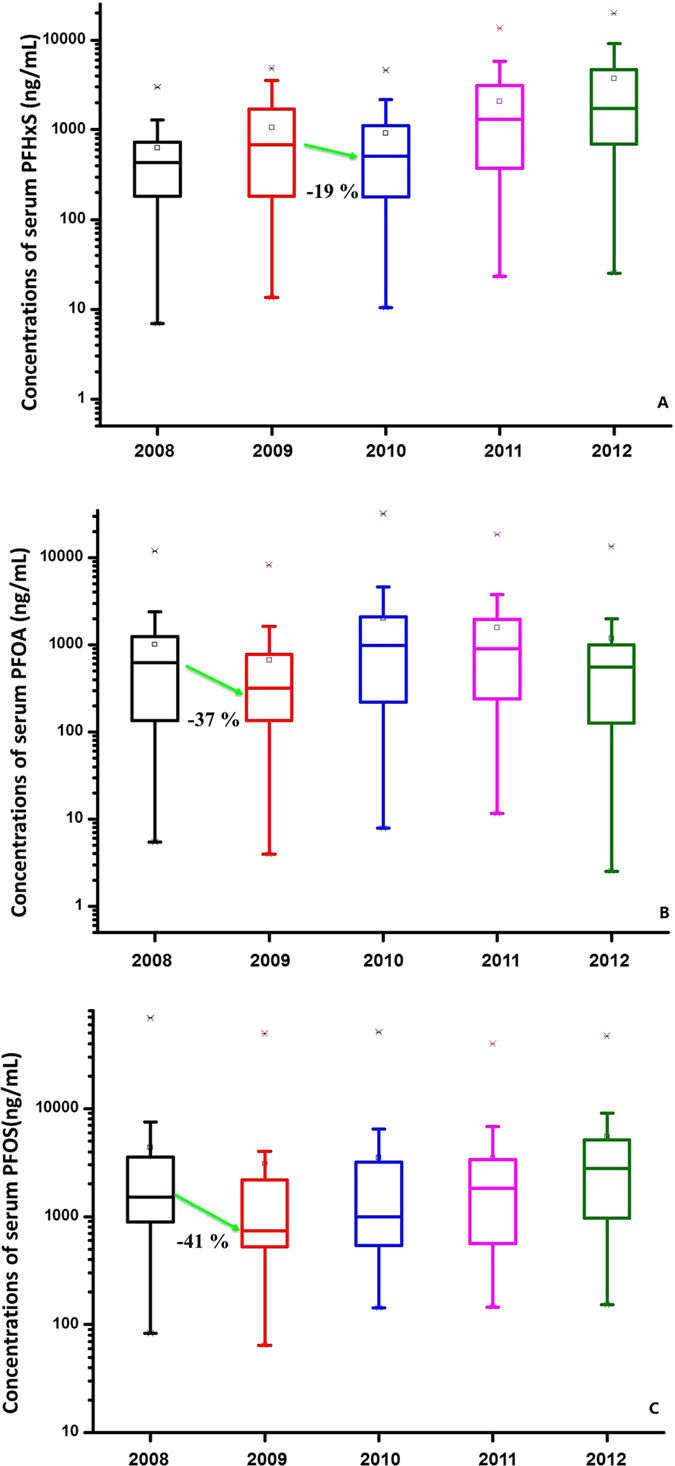
Temporal trends of PFAA serum concentrations in occupational workers from 2008 to 2012. The boxes represent 25^th^ and 75^th^ percentiles, and three horizontal bars represent the 5^th^, 50^th^, and 95^th^ percentiles; “×” denotes outliers. (**A**) PFHxS, (**B**) PFOA, (**C**) PFOS.

There was high employee turnover in this plant, and serum PFAA levels might relate to the working time. We selected workers who participated in our investigation at least three times during the five-year sampling period to better study the PFAA temporal trend in workers. 46 workers (41 in 2008, 38 in 2009, 43 in 2010, 42 in 2011, and 43 in 2012; the detailed information of the serum PFAA concentration is listed in Tables S3–S5) were selected to illustrate the PFAA temporal trend. The temporal trend for the body burden of PFAA in the 46 workers is shown in Fig. 1. The increase in the serum PFHxS levels in the 46 workers was highly significant between 2008 and 2012 (*p* < 0.01), and the GM of PFHxS presented a five-fold increase from 328 ng mL^−1^ in 2008 to 1673 ng mL^−1^ in 2012. The GM of the serum PFOS levels decreased from 1751 ng mL^−1^ in 2008 to 1095 ng mL^−1^ in 2009 and then doubled to 2580 ng mL^−1^ in 2012 (Fig. 1). The temporal trends of the PFAA body burden in the sampled workers closely relates to the product structure and total product volume of the plant. The annual productions of PFOS and PFHxS in the plant were approximately 60 and 0 tonnes in 2008, respectively. The output of PFOS was considerably reduced after PFOS was restricted in 2009 by the Stockholm Convention, and PFHxS then became a new product of the plant. The annual production volumes of PFOS from 2009 to 2011 were 30, 10, and 10 tonnes, respectively, whereas those of PFHxS were 10, 10 and 30 tonnes, respectively. The total annual PFAAs production volume was 60, 40, 20, 40, and 65 t between 2008 and 2012. The demand of PFAAs was reduced between 2009 and 2010 due to the severe economic crisis. The serum PFHxS decreased between 2009 and 2010 might due to the intermittent PFAAs production during that period. In 2012, the plant expanded the annual production of PFOS to 65 tonnes and ceased PFHxS production in light of changing market requirements, which led to the significant increase in the serum PFOS levels. Simultaneously, the synthesis of PFHxS-based fabric finishing agent continued in 2012 using the PFHxS in stock, thus resulting in a continual increase in the serum PFHxS levels. PFOA might be the byproduct in the electrolytic process, and no clear temporal trend was found for the serum PFOA. The GM of the serum PFOA was 368 ng mL^−1^ in 2008, 252 ng mL^−1^ in 2009, 622 ng mL^−1^ in 2010, 692 ng mL^−1^ in 2011, and 416 ng mL^−1^ in 2012.

## Discussion

According to the multi-way ANOVA analysis, the serum LnPFAAs were significantly different among sub-groups divided by gender (*p* < 0.05), length of service (*p* < 0.05), and work assignment (*p* < 0.05), whereas no significant difference was found among the sub-groups divided by BMI or age. A statistical description of the potential impact was made to evaluate the critical influencing factor of the PFAA body burden for the occupational workers. Specifically, a maximum value was observed for the coefficient of variance (CV) of the subgroup mean for work assignment, while the SD value for each subgroup was rather small. This implied that the work assignment might make a greater contribution to the variation in the LnPFAAs comparing to other variables. The CV of the subgroup mean for the length of service in this plant was second to that of work assignment.

**Figure 2:**
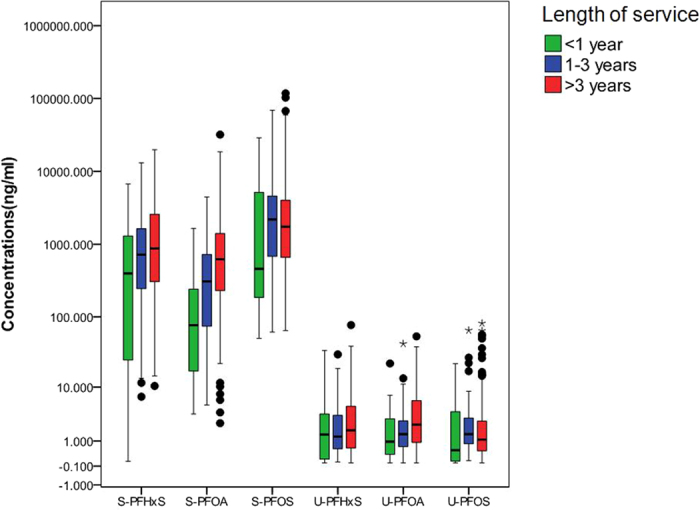
Serum PFAA concentrations among workers in the fluorochemical plant grouped by length of service in the plant.

Figure 2 illustrates the positive correlation between the serum PFAA levels and the service length of the corresponding workers, which indicates the prevailing role of the exposure time in the serum PFAA concentrations. Additionally, 3 tested PFAAs showed distinct exposure time *vs* body burden trends. The GMs of the serum PFOS, PFHxS and PFOA levels in the workers with service lengths less than 1 year were 782, 212 and 68 ng mL^−1^, respectively, while the GMs for PFOS steeply increased to 1861 ng mL^−1^ for those who have worked for 1–3 years. The further increase in the serum PFOS level is not significant for those who worked more than 3 years in the plant (1876 ng mL^−1^). On the other hand, the serum levels of PFHxS and PFOA in the exposed workers showed an obviously increasing trend with length of service, as the serum levels in the workers with service lengths between 1 and 3 years and more than 3 years were 521 and 222 ng mL^−1^ and 830 and 527, respectively. The PFAAs levels in different departments varied greatly, we further checked relationship between the serum PFAA levels and length of service in individual department, and the detailed information was listed in Table S6. Similar relationships were found between in the overall study population and in individual department. Such differences in the exposure time *vs* body burden trends may result from the accumulation kinetics of the pollutants. It seems that PFOS reaches a steady-state much faster than PFHxS and PFOA in humans. Andersen *et al*. found that under the condition of repeated-dose oral administration, PFOS approached the steady-state more rapidly than PFOA in *Cynomolgus* monkeys^36^. Moreover, in comparison with PFOA, PFOS had more binding sites and stronger affinities to human serum albumin^18^, which might accelerate the equilibration process of PFOS.

Our previous study suggested that urinary excretion is one of the primary clearance routes in rats for both PFOS and PFOA^37^, and the important role of renal clearance in human excretion was also proposed^28^,^29^,^38^. The positive correlations between the PFAAs in the serum and the paired urine samples were highly statistically significant, with correlation coefficients of 0.87, 0.64, and 0.72 for PFHxS, PFOA, and PFOS, respectively, (*p* < 0.01). Hence, the urine PFAA levels could be considered alternative bioindicators for the human PFAA body burden in occupational settings to some extent. Additionally, the relative abundances of PFOS, PFHxS, and PFOA between the serum and paired urine samples were quite different. The relative abundances in the serum are 64%, 24%, and 12%, respectively, while those in the urine were 19%, 31%, and 40%, respectively. The high relative abundance of PFOA in the urine displayed a faster elimination rate of PFOA than the rates of PFOS and PFHxS in humans. Moreover, the ratio of serum-to-urine concentrations (S/U) might be an indicator for PFAA excretion in humans because PFAAs could concentrate in serum while urine excretion is considered an important PFAA elimination pathway^29^. The GM values of S/U for PFHxS, PFOA, and PFOS in all samples were 697, 288, and 1782, respectively, indicating the difficulty in PFAA renal elimination from the workers. To exclude the potential effect of individual differences related to demographic information such as body weight and gender, the daily renal clearance rate (Cl_renal_) was introduced in the study. Cl_renal_ is a parameter defined in Eq (3) that adjusts the S/U through body weight and the gender-specific daily urine excretion volume.


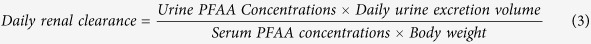


**Figure 3:**
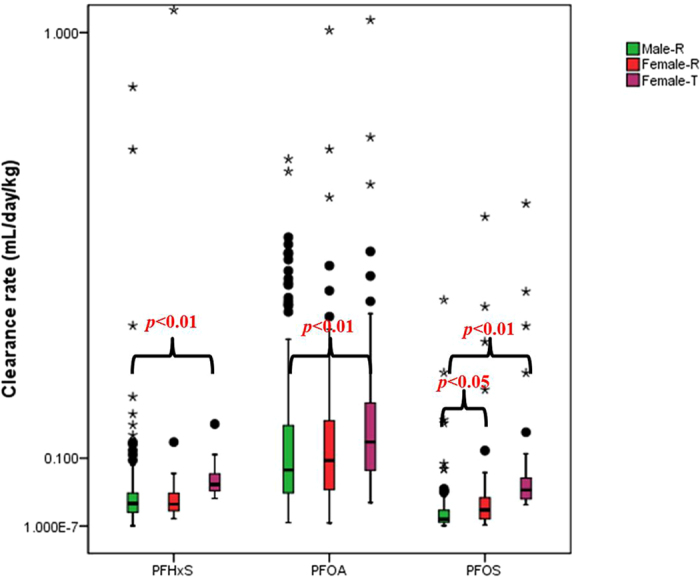
Clearance rates of PFAAs in males and females. Green, red, and purple boxes indicate the renal clearances of males and females and the total clearance of females, respectively. The boxes represent the 25^th^ and 75^th^ percentiles; the three horizontal bars represent the 5^th^, 50^th^, and 95^th^ percentiles; and “⚫” and “*” denotes outliers and extreme outliers, respectively.

Paired samples with either concentration under the LOD were set as LOD/2 in the Cl_renal_ study. The urine excretion volumes of males and females were set at 1.4 and 1.2 L/day in the study^28^, respectively, and the body weight values of individual workers were obtained from our questionnaires. The calculated Cl_renal_ values for PFHxS, PFOA, PFOS ranged from 2.0 × 10^−4^ to 2.3, 9.0 × 10^−5^ to 2.4, and 5.0 × 10^−5^ to 0.54 mL day^−1^ kg^−1^ with GM values of 0.023, 0.067 and 0.010 mL day^−1^ kg^−1^, respectively (Fig. 3). The minimum values of the PFAAs Cl_renal_ were calculated on the basis of LOD/2 instead of ND (not detected) in the present study. The Cl_renal_ estimates of PFOS were significantly lower than those of PFHxS in our study. However, Zhang *et al*. observed that the Cl_renal_ of PFHxS was the lowest among these 3 type of PFAAs^28^. The median Cl_renal_ of PFAAs in the present study was similar to that of Zhou *et al*.’s study (0.012 mL day^−1^ kg^−1^ for PFHxS, 0.061 mL day^−1^ kg^−1^ for PFOA, and 0.010 mL day^−1^ kg^−1^ for PFOS)^39^. In contrast, as noted elsewhere, the Cl_renal_ values of PFOA were the highest among those of the three PFAAs^28^,^38^. The most recent study on the elevated serum concentrations of PFAAs in fishery employees from Tangxun Lake also stated that the median Cl_renal_ of PFOA (0.061 mL day^−1^ kg^−1^) was higher than those of the PFHxS and PFOS (0.012 and 0.010 mL day^−1^ kg^−1^)^39^. It seemed that renal clearance was more efficient for PFOA than for PFOS and PFHxS, which resulted in the increase in the relative abundance of PFOA from 12% in the serum samples to 42% in the urine samples. Moreover, the renal clearance rates of PFAAs in humans were not obviously influenced by the PFAAs level in the serum. Although the reported body burdens of PFAAs varied by several orders of magnitude, the differences in the Cl_renal_ values of PFOA and PFOS obtained from different sources were not obvious, only diverse on the same order of magnitude (ranging from 0.01 to 0.1 mL day^−1^ kg^−1^)^28^,^39^,^40^, which suggested that Cl_renal_ was not correlated with the PFAA body burden. In addition, gender could not be discounted. The Cl_renal_ values of PFOS for the male workers were significant lower than those for the females (*p* < 0.01). Wong *et al*. studied the elimination rate constant of PFOS in a USA population and speculated that there might be other sex-specific elimination routes other than menstruation between men and women^41^. No significant differences were found between the Cl_renal_ values of PFOA and PFHxS for the males and females tested in the study.

Renal clearance was assumed to be the major elimination pathway of PFAAs for males and females older than 50^28^,^37^,^40^, but we need to note that menstrual clearance is another important excretion route of PFAAs for females under 50 that is comparable to renal clearance. The reported average menstrual clearance rate of 0.029 mL day^−1^ kg^−1^ in young females^40^ and the daily renal clearance rates of the PFAAs (GM: 0.024, 0.062 and 0.017 mL day^−1^ kg^−1^ for PFHxS, PFOA, and PFOS, respectively) had the same order of magnitude. We used a first order model to estimate the half-lives of PFAAs in workers (Eq (2)). *k* was defined as V_d_/Cl_total_, where Cl_total_ referred to renal clearance for men and women older than 50 and to the sum of menstrual clearance and renal clearance for young women. According to Eq (2), the half-lives of PFHxS, PFOA, and PFOS for the occupational workers tested ranged from 0.51–3799 years, 0.44–3663 years, and 0.76–30475 years, with GM and median values of 14.7 and 11.7, 4.1 and 4.0, 32.6 and 21.6 years, respectively (Table 2). Naturally, the female workers showed significantly higher clearance rate (p < 0.01) than the males in the present study if the menstrual clearance for females under 50 was taken into consideration when calculating the total clearance rate (Cl_total_) (Fig. 3). This resulted in significantly longer half-lives of PFHxS, PFOA, and PFOS in male workers relative to those of the females (*p* < 0.01). The GM values of the half-lives of PFHxS, PFOA, and PFOS for men here were 19.9, 4.7, and 60.9 years, respectively, while those for women were 7.5, 3.1, and 8.0 years, respectively. The half-lives estimated by the limited clearance route information could be considered as the upper limits for PFAAs. It seemed likely that the unrealistically long half-life estimates derived from the urinary data were actually evidence of the contribution of fecal elimination to the clearance of these chemicals. The extremely long half-lives of PFAAs, such as 3799 years for PFHxS, 3663 years for PFOA, and 30475 years for PFOS, were caused by the application of LOD/2 instead of ND in workers’ urine samples. The geometric mean or median value of the PFAAs half-lives can better reflect the actual situation.

Serum PFAAs of workers were sequentially measured for 5 years, and they fluctuated during our sampling period, although different serum PFAAs decreased in certain years. The serum PFHxS concentration in workers declined from 455 ng mL^−1^ in 2009 to 366 ng mL^−1^ in 2010, and 24 out of 37 workers had a decrease in serum PFHxS, corresponding to an annual drop of 19%. For PFOA, 31 out of 42 workers decreased in the period of 2011–2012, and the serum PFOA concentration dropped by 41%. 36 workers participated in the sampling activities in both 2008 and 2009, and the serum PFOS declined in 33 out of 36 workers, with 7 out of 36 workers exhibiting a dramatic decrease of more than 50%, and the average annual decline rate reached 37% (Fig. 1). We assumed that there were no new inputs of PFAAs in these workers, and thus *k* in Eq (2) was set as the average annual serum PFAA decline rate. The half-lives of PFHxS, PFOA, and PFOS estimated by Eq (2) were 3.6, 1.7, and 1.9 years. Though only two time points included in the estimation, the rapid decrease of PFAAs in workers suggested the underestimated elimination potential of PFAAs in previous studies^21^,^28^,^41^.

Zhang *et al*. estimated the half-lives of PFAAs by daily clearance and found that the half-lives of PFHxS, PFOA, and PFOS in young females were 7.8 1.5 and 5.1 years (GM), respectively, and in the males and older females were 25, 1.2, and 18 years (GM), respectively^28^. However, the half-lives of PFAAs derived from the longitudinal data or cross-sectional data were much shorter than those from the clearance pathways. Olsen *et al*. reported that the half-lives of PFHxS, PFOA, and PFOS were 7.3, 3.5, and 4.8 years (GM), respectively, in retired fluorochemical production workers^21^. Nevertheless, a population-based pharmacokinetic model based on six cross-sectional data sets from 1999 to 2012 from the US National Health and Nutrition Examination Survey reported the half-life of PFOS in humans to be 3.7 years^41^. Specifically, the shortest half-lives of PFHxS, PFOA and PFOS in humans reported in previous studies were 7.3, 1.2 and, 3.7 years, respectively^21^,^28^,^41^. Fu *et al*. estimated that the daily PFAA ingestion of workers reached 2600 ng/kg/day, and the ongoing exposure of the workers to PFAAs was inevitable^35^. High ongoing levels of PFAAs exposure could give a false impression of longer half-lives^42^,^43^, and the half-lives of PFHxS, PFOA, and PFOS derived from annual decline rates in the present study still longer than the intrinsic half-lives of PFAAs in occupational workers. To our knowledge, the half-life values for PFOS and PFHxS are the shortest reported so far, merely half of the previously reported values. This introduces an interesting question essential to the toxicity of PFAAs: Are the intrinsic half-lives of PFAAs in human shorter than expected? According to the result of our investigation, the clearance capacity of PFOS and PFHxS in humans is likely underestimated. Correspondingly, the half-lives of the three PFAAs for the general population with rather low serum contamination levels need further study.

The half-life of PFHxS was shorter than that of PFOS estimated by daily clearance, however, it was longer than PFOS by using annual decline rates in the present study. Moreover, a shorter half-life for PFOS was reported by both Olsen and Zhang compared with PFHxS^21^,^28^. The reason for such an inconsistency remains unclear. Noker and Gorman found that the half-life of PFHxS was similar to that of PFOS in monkeys^44^,^45^, and a rodent test using Sprague-Dawley rats found that the half-life of PFHxS was shorter than that of PFOS during a single dose exposure^46^. As an alternative to PFOS, PFHxS should be addressed with respect to its clearance and half-life in humans.

The half-life of PFOA was shorter than that of PFHxS and PFOS, which was consistent with previous studies^21^,^28^. Nevertheless, the half-life of PFOA presented a slight deviation from those of the two referenced studies^21^,^28^, and the possible reason may be the difference in the elimination mechanisms between PFOA and PFSA in humans. Renal clearance might be the most predominant excretion route for PFOA. Lupton *et al*. found that PFOA was mainly eliminated by urine in cattle, while PFOS was minimally eliminated through urine and it could be transported to the bile and then eliminated in feces or reabsorbed by the intestinal tract^47^,^48^. A previous study also implied that the biliary excretion rate of PFOA was higher than that of PFOS due to the higher reabsorption rate of PFOS through the enterohepatic circulation process in humans^30^. The higher reabsorption rate of PFOS in both the enterohepatic circulation process and the renal tubular reabsorption partly explains the reason for the longer half-life of PFOS compared to that of PFOA^36^.

There are several limitations in the half-lives of PFAAs in the present study: 1) too many variables can affect PFAAs concentration in urine, ingestion of a lot of water right before a test could dilute the sample; Aimed at this limitation, we collected morning urine samples of workers before breakfast to minimize the errors between individuals. 2) the clearance pathways of PFSAs in humans are not limited to renal clearance and/or menstruation; Excretion routes other than urine and menstruation also play important roles in the elimination of PFHxS and PFOS in humans, and the half-lives estimated by clearance rates of PFAAs could be considered as upper limits. 3) the apparent half-lives of PFAAs calculated through annual decline rates could be affected by the high ongoing levels of exposure^42^,^43^. The half-lives of PFHxS and PFOS estimated by annual decline rates were much shorter than estimated by daily clearance rates and previously reported, which indicated that there is other important elimination pathway of PFHxS and PFOS other than renal clearance, and the elimination potential of PFAAs might have been underestimated. If considering influence of ongoing exposure to PFAAs in workers, the intrinsic half-lives of PFAAs were even shorter. Blood and urine clinical chemistry data of the workers were also measured (Table S7), though these parameters were not significant different between workers and normal human, an awareness of the risk caused by the relevant exposure should be raised.

## Additional Information

**How to cite this article**: Fu, J. *et al*. Occurrence, temporal trends, and half-lives of perfluoroalkyl acids (PFAAs) in occupational workers in China. *Sci. Rep.* **6**, 38039; doi: 10.1038/srep38039 (2016).

**Publisher's note:** Springer Nature remains neutral with regard to jurisdictional claims in published maps and institutional affiliations.

## Supplementary Material

Supplementary Information

## Figures and Tables

**Table 1:** Descriptive statistics of PFAAs in serum and urine samples (all workers, divided by working location, ng mL^−1^).

Group	N		Serum			N		Urine		
			**PFHxS**	**PFOA**	**PFOS**			**PFHxS**	**PFOA**	**PFOS**
All workers										
All workers	302	N > LOD	301	302	302	274	N > LOD	245	254	249
		mean	1855	1052	5624		mean	3.9	4.3	4.4
		median	764	427	1725		median	1.7	1.9	1.2
		range	LOD-19837	2.5–32000	50.3–118000		range	LOD-77.1	LOD-53.6	LOD-81.5
Divide by work assignments										
Electrolytic department	74	N > LOD	74	74	74	67	N > LOD	66	65	64
		mean	1469	2337	1909		mean	2.3	6.7	1.8
		median	1011	1126	1541		median	1.3	3.5	0.93
		range	46.6–6759	55.9–32000	234–8501		range	LOD-8.3	LOD-38.4	LOD-26.9
Sulfonation department	101	N > LOD	101	101	101	98	N > LOD	95	97	96
		mean	3778	929	14002		mean	7.1	4.8	8.8
		median	2250	603	5544		median	3.7	2.7	3.0
		range	96.1–15700	4.9–4630	416–118000		range	LOD-77.1	LOD-53.6	LOD-81.5
Research building	27	N > LOD	27	27	27	25	N > LOD	21	23	21
		mean	842	404	1195		mean	1.9	1.8	1.4
		median	506	142	736		median	0.97	0.92	1.1
		range	3.0–3360	4.7–2920	101–7450		range	LOD-7.6	LOD-15.3	LOD-6.5
Fabric finishing agent department	8	N > LOD	8	8	8	8	N > LOD	8	8	4
		mean	1956	606	514		mean	3.5	5.1	0.39
		median	1768	114	267		median	2.4	1.5	LOD
		range	550–4690	51–2600	103–1890		range	0.79–7.1	1.2–22.2	LOD-1.7
Management office	92	N > LOD	91	92	92	76	N > LOD	55	61	64
		mean	331	362	1144		mean	0.93	1.5	1.6
		median	169	101	616		median	0.37	0.86	0.49
		range	LOD-3350	2.5–4380	50.3–7910		range	LOD-8.1	LOD-9.7	LOD-12.1

**Table 2:** Half-lives of PFAAs in workers estimated through daily elimination (menstrual clearance added to renal clearance for females).

	number		PFHxS (years)	PFOA (years)	PFOS (years)
Half-lives of PFAAs estimated through daily elimination					
Male	136	Min	0.51	0.48	2.7
		Max	3799	3663	30475
		Median	14.5	4.4	49.3
		GM	19.9	4.7	60.9
		GSD	3.9	3.0	4.8
Female	71	Min	2.8	0.44	0.76
		Max	15.1	11.1	15.1
		Median	7.6	2.8	9.4
		GM	7.5	3.1	8.0
		GSD	1.4	2.2	1.9
Total	207	Min	0.51	0.44	0.76
		Max	3799	3663	30475
		Median	11.7	4.0	21.6
		GM	14.7	4.1	32.6
		GSD	3.4	2.8	5.1
