# Supplementary material for: Occurrence, temporal trends, and half-lives of perfluoroalkyl acids (PFAAs) in occupational workers in China
Source: Sci Rep. 2016 Dec 1;6:38039. doi: 10.1038/srep38039 (PMC5131319; doi:10.1038/srep38039)
Supplement: Supplementary Information [file srep38039-s1.doc]

Supplementary Information

# Occurrence, temporal trends, and half-lives of perfluoroalkyl acids (PFAAs) in occupational workers in China

Jianjie Fu^1^, Yan Gao^1^, Lin Cui^1^, Thanh Wang^1^, Yong Liang^2,3^, Guangbo Qu^1^, Bo Yuan^1^, Yawei Wang*^1^, Aiqian Zhang*^1,2^, and Guibin Jiang^1^

^1^State Key Laboratory of Environmental Chemistry and Ecotoxicology, Research Center for Eco-Environmental Sciences, Chinese Academy of Sciences, Beijing 100085, China

^2^Institute of Environment and health, Jianghan University

^3^ School of Medicine, Jianghan University, Wuhan 430056, China

*Corresponding author

Prof. Aiqian Zhang

Tel: +86 - 10 - 62849157

Fax: +86 - 10 - 62923549

E - mail: [aqzhang@rcees.ac.cn](mailto:aqzhang@rcees.ac.cn)

Dr. Yawei Wang

Tel: +86 - 10 - 6284 - 9334

Fax: +86 - 10 - 6284 - 9339

E - mail: [ywwang@rcees.ac.cn](mailto:ywwang@rcees.ac.cn)

Research Center for Eco - Environmental Sciences,

Chinese Academy of Sciences, Beijing 100085, China

**Sample pretreatment and instrument analysis**

Briefly, serum samples were pretreated by ion-pairing extraction and solid phase extraction. An Oasis - HLB cartridge (Waters, 6 cc, 150 mg) was preconditioned with 7 mL methanol followed by 7 mL of water. Approximately 0.01 - 0.4 mL of the serum samples was added into a 15 mL polypropylene (PP) tube and spiked with 5 ng of internal standard. Subsequently, 1 mL of 0.5 M TBA, 2 mL of 0.25 M sodium carbonate buffer (pH=10) and 5 mL of MTBE were added. The mixture was shaken at 270 rpm for 20 min and centrifuged at 3500 rpm for 10 min. The supernatant was transferred into a new PP tube. The extraction was repeated another two times, and the organic layers were combined. The combined organic solution was concentrated to 2 - 3 mL under gentle nitrogen gas, and 1 mL methanol was added. The mixture was finally concentrated to 0.5 mL and then diluted with 50 mL water. The dilution was loaded onto the preconditioned Oasis-HLB cartridge, after which 5 mL of methanol/water (1:4) was loaded. Target analytes were eluted by 10 mL methanol. The final elution was concentrated to 1 mL before injection into the HPLC - ESI/MS/MS.

Urine samples were pretreated by solid phase extraction. Briefly, 1 mL of urine spiked with 5 ng of internal standard was added to a 15 - mL PP tube and diluted with 13 mL water. The solid phase extraction procedure was then performed following the previously described serum extraction procedure.

Analysis of PFAAs was performed using a HPLC-ESI/MS/MS system. A 10 - µL sample solution was injected into a Dionex Acclaim 120 C_18_ column (5 μm, 4.6 mm i.d.×150 mm length; Dionex, Sunnyvale, CA, USA) HPLC-ESI/MS/MS system. Methanol (A) and 50 mM ammonium acetate (B) were used as the mobile phase solvents. The flow rate was 1 mL/min. A dualistic gradient was performed as follows: initial at 28% B, then 5% B in 4 min, returned to 28% B in 3 min, and then equilibrated for 3 min before the next injection.

**Quality assurance and quality control**

^13^C_4_ - PFOS and ^13^C_4_ - PFOA were used as internal standards. PFAA standards were added into the serum samples at the 25 ng (n=3) and 50 ng (n=3) levels, and the recoveries of PFHxS, PFOS, and PFOA ranged from 106±1.4% to 110 ± 4.2%, 98.1 ± 2.4% to 101 ± 3.8%, and from 94.9 ± 4.4% to 96.6 ± 3.1%, respectively. Urine samples were spiked with 10 ng standards (n=6), and the recoveries of PFHxS, PFOS, and PFOA were 123 ± 6.0%, 114 ± 3.9%, and 111 ± 3.6%. A procedural blank was performed for every batch of seven samples, and all blank samples were below the LODs.


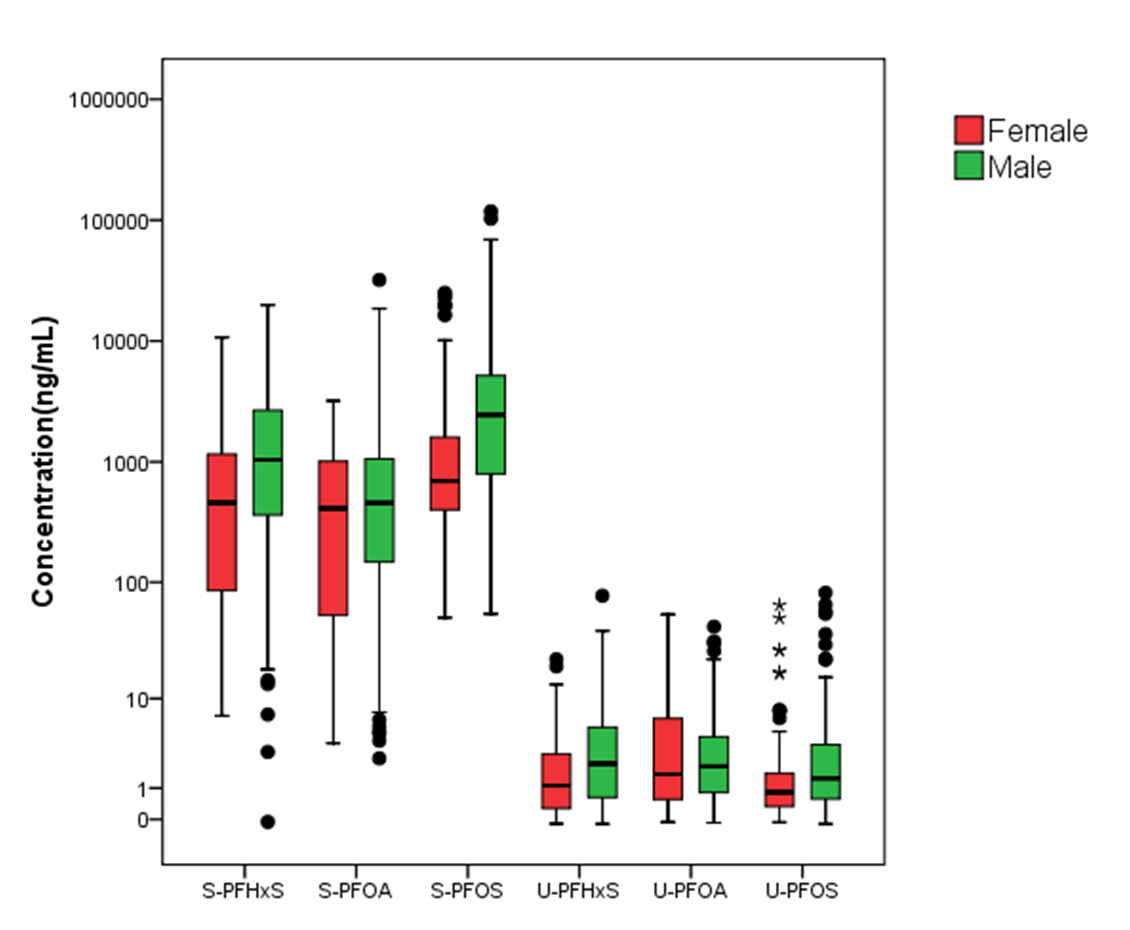


**Figure S1** PFAA concentrations in serum (S) and urine (U) samples compared between male and female subjects during the period of 2008-2012. Single values beyond the 150^th^ percentiles of the difference between the 25^th^ and 75^th^ percentiles are denoted by black circles, whereas extreme values are denoted by “*”, which are beyond triple the difference between the 25^th^ and 75^th^ percentiles.

**Table S1** Descriptive statistics of the demographic information

|  |  | Min | Max | Median | Average | SD |
| --- | --- | --- | --- | --- | --- | --- |
|  | Height (cm) | 150 | 184 | 165 | 165 | 7 |
| Total | Weight (kg) | 43 | 90 | 60 | 62 | 11 |
|  | BMI (kg/m^2^) | 16.6 | 30.0 | 22.6 | 22.6 | 2.6 |
|  | Age | 19 | 65 | 41 | 41 | 10 |
|  |  |  |  |  |  |  |
| Female | Height (cm) | 150 | 167 | 160 | 164 | 4 |
|  | Weight (kg) | 43 | 75 | 55 | 62 | 8 |
|  | BMI (kg/m^2^) | 16.6 | 30.0 | 20.7 | 21.6 | 2.9 |
|  | Age | 19 | 50 | 37 | 37 | 8 |
|  |  |  |  |  |  |  |
| Male | Height (cm) | 154 | 184 | 170 | 169 | 5 |
|  | Weight (kg) | 52 | 90 | 68.5 | 68 | 9 |
|  | BMI (kg/m^2^) | 18.0 | 29.7 | 23.0 | 23.0 | 2.4 |
|  | Age | 22 | 65 | 43 | 43 | 10 |

**Table S2** Spearman’s correlation coefficients of serum PFAAs in different sub-groups.

| All workers | | | |
| --- | --- | --- | --- |
|  | PFHxS | PFOA | PFOS |
| PFHxS | 1.000 | 0.756^**^ | 0.615^**^ |
| PFOA | 0.756^**^ | 1.000 | 0.521^**^ |
| PFOS | 0.615^**^ | 0.521^**^ | 1.000 |
| Sulfonation department | | | |
|  | PFHxS | PFOA | PFOS |
| PFHxS | 1.000 | 0.338^**^ | 0.567^**^ |
| PFOA | 0.338^**^ | 1.000 | 0.092 |
| PFOS | 0.567^**^ | 0.092 | 1.000 |
| Electrolytic department | | | |
|  | PFHxS | PFOA | PFOS |
| PFHxS | 1.000 | 0.432^**^ | 0.548^**^ |
| PFOA | 0.432^**^ | 1.000 | 0.369^**^ |
| PFOS | 0.548^**^ | 0.369^**^ | 1.000 |
| Management office | | | |
|  | PFHxS | PFOA | PFOS |
| PFHxS | 1.000 | 0.816^**^ | 0.802^**^ |
| PFOA | 0.816^**^ | 1.000 | 0.689^**^ |
| PFOS | 0.802^**^ | 0.689^**^ | 1.000 |
| Fabric finishing agent department | | | |
|  | PFHxS | PFOA | PFOS |
| PFHxS | 1 | 0.452 | 0.833* |
| PFOA | 0.452 | 1 | 0.762* |
| PFOS | 0.833* | 0.762* | 1 |
| Research building | | | |
|  | PFHxS | PFOA | PFOS |
| PFHxS | 1.000 | 0.460^*^ | 0.664^**^ |
| PFOA | 0.460^*^ | 1.000 | 0.381^*^ |
| PFOS | 0.664^**^ | 0.381^*^ | 1.000 |

**. Correlation is significant at the 0.01 level (2-tailed).

*. Correlation is significant at the 0.05 level (2-tailed).

**Table S3** Serum and urine PFHxS concentrations, renal clearance rate in 46 workers who participated in our investigation at least three times during the five-year sampling period.

| No. | Gender | Department | Serum (ng/mL) | | | | | Urine (ng/mL) | | | | | Renal clearance rate(mL/day/kg) | | | | |
| --- | --- | --- | --- | --- | --- | --- | --- | --- | --- | --- | --- | --- | --- | --- | --- | --- | --- |
|  |  |  | 2008 | 2009 | 2010 | 2011 | 2012 | 2008 | 2009 | 2010 | 2011 | 2012 | 2008 | 2009 | 2010 | 2011 | 2012 |
| 1 | Female | E^a^ | 183 | 1080 | 772 | 3220 | 3792 |  |  | 1.84 | 6.30 | 6.59 |  |  | 0.064 | 0.050 | 0.042 |
| 2 | Female | E | 331 | 877 | 718 | 1402 | 3428 | 0.939 |  | 1.02 | 2.55 | 3.58 | 0.061 |  | 0.028 | 0.036 | 0.021 |
| 3 | Female | E | 443 | 356 | 192 | 174 | 277 |  |  | 0.292 |  | 0.98 |  |  | 0.024 |  | 0.061 |
| 4 | Female | E | 731 | 790 | 540 | 2230 | 1549 | 1.05 | 1.12 | 1 | 8.28 | 1.42 | 0.028 | 0.027 | 0.037 | 0.074 | 0.018 |
| 5 | Female | E | 628 | 971 | 459 | 2185 | 3566 | 1.3 | 1.18 | 0.366 | 5.30 | 7.64 | 0.044 | 0.026 | 0.016 | 0.050 | 0.044 |
| 6 | Female | E | 641 | 1380 | 1110 | 2290 | -- | 1.1 | 1.33 | 1.56 | 3.88 |  | 0.039 | 0.022 | 0.031 | 0.037 |  |
| 7 | Female | E | 434 | --^b^ | 202 | 1305 | 2589 | 0.616 |  | 0.254 | 2.09 | 4.54 | 0.025 |  | 0.023 | 0.028 | 0.031 |
| 8 | Female | A | 31 | 43 | 37 | 85 | 276 | 0.0676 |  | 0.117 |  |  | 0.051 |  | 0.077 |  |  |
| 9 | Female | A | -- | 13.5 | 10.4 | 23.2 | 60.0 |  | 0.075 |  |  |  |  | 0.125 |  |  |  |
| 10 | Female | A | 6.9 | 32.6 | 53.9 | -- | 263 | 0.0162 |  |  |  |  | 0.051 |  |  |  |  |
| 11 | Female | A | 18.4 | -- | 11.6 | 73.3 | 185 | 0.0311 |  |  |  |  | 0.047 |  |  |  |  |
| 12 | Female | A | 34.4 | 24.9 | 21.2 | -- | -- | 0.0451 | 0.0197 |  |  |  | 0.034 | 0.021 |  |  |  |
| 13 | Female | S | 842 | 1400 | 1480 | 8020 | 10722 | 1.33 | 1.42 | 3.12 | 22.20 | 19.3 | 0.033 | 0.021 | 0.044 | 0.057 | 0.035 |
| 14 | Female | S | 606 | 2250 | 1480 | 2430 | 4326 |  | 4.43 | 1.4 | 3.74 | 5.92 |  | 0.041 | 0.019 | 0.031 | 0.027 |
| 15 | Female | S | 1710 | 3550 | 4600 | 13500 | 10845 | 3.04 | 4.97 | 6.4 | 26.30 | 39.0 | 0.043 | 0.034 | 0.032 | 0.045 | 0.082 |
| 16 | Female | R | 83 | 244 | 233 | 500 | 1088 | 0.0377 |  | 0.287 |  | 0.92 | 0.010 |  | 0.027 |  | 0.019 |
| 17 | Female | R | 364 | 635 | 747 | 830 | 1650 |  | 0.352 | 0.385 | 0.75 | 1.08 |  | 0.013 | 0.012 | 0.022 | 0.016 |
| 18 | Male | E | 1150 | 3150 | 3940 | 5836 | 4690 | 0.667 | 4.66 | 3.84 | 7.12 | 6.02 | 0.015 | 0.038 | 0.025 | 0.036 | 0.024 |
| 19 | Male | E | 333 | 1960 | 1840 | 3115 | 6759 | 0.488 |  | 1.71 | 5.37 | 7.88 | 0.037 |  | 0.022 | 0.040 | 0.026 |
| 20 | Male | E | 2070 | 2600 | 2170 | 4260 | 3544 | 1.79 | 1.83 | 1.38 | 3.46 | 6 | 0.017 | 0.014 | 0.012 | 0.016 | 0.034 |
| 21 | Male | E | 719 | 733 | 756 | 970 | 1326 | 0.937 | 1.24 | 0.735 | 0.54 | 3.96 | 0.028 | 0.046 | 0.026 | 0.015 | 0.080 |
| 22 | Male | E | -- | -- | 47 | 372 | 1424 |  |  | 0.16 | 0.06 | 2.32 |  |  | 0.064 | 0.003 | 0.027 |
| 23 | Male | A | 722 | 756 | 586 | 687 | 1755 | 1.52 | 0.679 | 2.27 | 0.32 | 0.94 | 0.044 | 0.018 | 0.077 | 0.010 | 0.011 |
| 24 | Male | A | 150 | 182 | 166 | 794 | 878 | 0.326 |  | 0.042 | 0.84 | 1.94 | 0.049 |  | 0.005 | 0.022 | 0.048 |
| 25 | Male | A | 192 | 181 | 139 | 241 | 385 | 0.381 | 0.275 | 0.080 |  | 0.18 | 0.030 | 0.023 | 0.009 |  | 0.007 |
| 26 | Male | A | 245 | 163 | 193 | 183 | 165 |  | 0.142 |  |  | 0.52 |  | 0.017 |  |  | 0.063 |
| 27 | Male | A | 412 | 370 | 318 | 281 | 429 | 0.513 | 0.236 |  | 0.05 | 0.68 | 0.024 | 0.012 |  | 0.004 | 0.031 |
| 28 | Male | A | 422 | 292 | 353 | 738 | 1311 | 0.596 | 0.435 | 0.5 |  | 2.76 | 0.033 | 0.035 | 0.032 |  | 0.049 |
| 29 | Male | A | 7.1 | 25.4 | 19.1 | 27.4 | 25.3 | 0.011 | 0.050 |  |  | 1.2 | 0.030 | 0.039 |  |  | 0.853 |
| 30 | Male | A | 242 | 219 | 215 | 230 | 690 | 0.296 | 0.406 |  |  | 3.74 | 0.024 | 0.037 |  |  | 0.105 |
| 31 | Male | A | 91.4 | 90.2 | -- | 172 | 411 | 0.379 | 0.188 |  |  |  | 0.097 | 0.049 |  |  |  |
| 32 | Male | A | 444 | -- | 362 | 400 | 1380 | 0.668 |  | 0.51 | 0.37 | 8.06 | 0.035 |  | 0.036 | 0.022 | 0.136 |
| 33 | Male | A | 147 | 20.1 | 14.6 | -- | -- | 0.0314 |  |  |  |  | 0.004 |  |  |  |  |
| 34 | Male | S | 1740 | 2540 | 2000 | 4390 | 6173 | 2.44 | 2.94 | 5.63 | 5.00 | 8.62 | 0.030 | 0.029 | 0.064 | 0.027 | 0.032 |
| 35 | Male | S | 746 | 1700 | 1040 | 3950 | 19837 | 0.764 | 2.17 | 5.07 | 9.29 | 9.54 | 0.020 | 0.034 | 0.090 | 0.047 | 0.010 |
| 36 | Male | S | 643 | 969 | 743 | 1920 | 1686 | 1.26 | 0.676 | 2.67 | 1.85 | 5.48 | 0.038 | 0.013 | 0.067 | 0.018 | 0.057 |
| 37 | Male | S | 1070 | 635 | 1130 | 1838 | 1896 | 0.381 | 0.127 | 1.76 | 2.22 | 3.67 | 0.008 | 0.005 | 0.036 | 0.028 | 0.045 |
| 38 | Male | S | 436 | 549 | 648 | 2100 | 6489 | 0.831 | 0.091 | 4.24 | 3.26 | 13.5 | 0.044 | 0.004 | 0.153 | 0.036 | 0.049 |
| 39 | Male | S | 1280 | 2440 | 2600 | 3970 | 13288 |  | 3.54 | 2.9 | 5.88 | 77.1 |  | 0.030 | 0.024 | 0.032 | 0.125 |
| 40 | Male | S | -- | 4860 | 3910 | 5800 | 13429 |  | 4.2 | 1.91 | 9.93 | 12.8 |  | 0.018 | 0.010 | 0.037 | 0.020 |
| 41 | Male | S | 719 | -- | 719 | 1800 | 4601 | 0.567 |  | 0.767 | 2.36 | 2.94 | 0.020 |  | 0.027 | 0.032 | 0.016 |
| 42 | Male | S | 1580 | -- | -- | 790 | 8163 |  |  |  | 2.71 | 11.0 |  |  |  | 0.074 | 0.030 |
| 43 | Male | S | -- | -- | 471 | 1550 | 3137 |  |  | 1.18 | 4.12 | 4.74 |  |  | 0.047 | 0.051 | 0.029 |
| 44 | Male | R | 137 | 219 | 459 | 606 | 1116 | 0.357 | 1.43 | 0.972 | 6.46 | 1.7 | 0.048 | 0.122 | 0.037 | 0.199 | 0.027 |
| 45 | Male | R | 2970 | 1750 | 2670 | 3240 | 9194 | 3.38 | 3.74 | 6.61 | 3.05 | 12.7 | 0.022 | 0.042 | 0.050 | 0.018 | 0.026 |
| 46 | Male | R | -- | -- | 68.6 | 506 | 1734 |  |  |  |  | 2.2 |  |  |  |  | 0.025 |

a: Work assignment, E, A, R, S here represent the electrolytic process department, management office, research and development department, and sulfonation department, respectively. b: Data not available, the worker did not participate in sampling that year.

**Table S4** Serum and urine PFOA concentrations, renal clearance rate in 46 workers who participated in our investigation at least three times during the five-year sampling period.

| No. | Gender | Department | Serum (ng/mL) | | | | | Urine (ng/mL) | | | | | Renal clearance rate(mL/day/kg) | | | | |  |
| --- | --- | --- | --- | --- | --- | --- | --- | --- | --- | --- | --- | --- | --- | --- | --- | --- | --- | --- |
|  |  |  | 2008 | 2009 | 2010 | 2011 | 2012 | 2008 | 2009 | 2010 | 2011 | 2012 | 2008 | 2009 | 2010 | 2011 | 2012 |  |
| 1 | Female | E^a^ | 69 | 199 | 1410 | 1490 | 550 |  |  | 1.09 | 10.70 | 2.5 |  |  | 0.021 | 0.183 | 0.109 |  |
| 2 | Female | E | 331 | 877 | 718 | 1402 | 3428 | 5 |  | 0.579 | 10.80 | 1.74 | 0.091 |  | 0.004 | 0.145 | 0.082 |  |
| 3 | Female | E | 1820 | 1640 | 2440 | 1300 | 234 |  |  | 1.08 |  | 1.32 |  |  | 0.007 |  | 0.097 |  |
| 4 | Female | E | 1270 | 630 | 2430 | 1960 | 676 | 8.09 | 19.1 | 0.61 | 38.40 | 0.42 | 0.123 | 0.587 | 0.005 | 0.392 | 0.012 |  |
| 5 | Female | E | 1250 | 833 | 2060 | 900 | 679 | 7.15 | 10 | 1.1 | 8.54 | 1.44 | 0.123 | 0.257 | 0.011 | 0.196 | 0.044 |  |
| 6 | Female | E | 1400 | 785 | 3200 | 2330 | -- | 6.68 | 4.81 | 1.12 | 9.15 |  | 0.108 | 0.139 | 0.008 | 0.086 |  |  |
| 7 | Female | E | 988 | --^b^ | 1990 | 1395 | 597 | 5.18 |  | 1.15 | 7.96 | 3 | 0.093 |  | 0.010 | 0.101 | 0.089 |  |
| 8 | Female | A | 49 | 48 | 112 | 94 | 53 | 0.0901 |  | 0.394 | 0.80 |  | 0.043 |  | 0.084 | 0.205 |  |  |
| 9 | Female | A | 12.1 | 30.7 | 22.2 | 39.6 | 61.5 |  |  | 0.234 |  |  |  |  | 0.173 |  |  |  |
| 10 | Female | A | 6.8 | 5.3 | 22.9 | -- | 41.6 |  |  |  |  |  |  |  |  |  |  |  |
| 11 | Female | A | 18.5 | -- | 30.8 | 48.1 | 70.0 | 0.0937 |  | 0.51 |  |  | 0.141 |  | 0.442 |  |  |  |
| 12 | Female | A | 62.4 | 28.1 | 75.2 | -- | -- | 0.178 |  | 0.0794 |  |  | 0.074 |  | 0.028 |  |  |  |
| 13 | Female | S | 661 | 411 | 1590 | 1010 | 718 | 2.21 | 3.45 | 53.6 | 6.69 | 1.89 | 0.069 | 0.174 | 0.697 | 0.137 | 0.052 |  |
| 14 | Female | S | 982 | 573 | 1410 | 662 | 458 |  | 8.82 | 4.78 | 4.04 | 1.2 |  | 0.318 | 0.068 | 0.124 | 0.052 |  |
| 15 | Female | S | 2120 | 1250 | 4630 | 3290 | 4481 | 7.6 | 14.8 | 1.06 | 11.1 | 7.38 | 0.087 | 0.286 | 0.005 | 0.077 | 0.038 |  |
| 16 | Female | R | 91 | 49 | 143 | 168 | 93 | 0.0489 |  | 0.919 |  | 0.22 | 0.012 |  | 0.143 |  | 0.052 |  |
| 17 | Female | R | 849 | 318 | 923 | 838 | 266 |  | 1.48 | 1.28 | 3.36 | 0.5 | 0.000 | 0.110 | 0.033 | 0.096 | 0.045 |  |
| 18 | Male | E | 1470 | 1100 | 4210 | 1094 | 2600 | 3.48 | 15.8 | 0.775 | 8.89 | 3.84 | 0.060 | 0.366 | 0.005 | 0.081 | 0.082 |  |
| 19 | Male | E | 750 | 474 | 3560 | 3805 | 6181 | 3.75 |  |  | 30.8 | 31.6 | 0.127 |  | 0.000 | 0.189 | 0.114 |  |
| 20 | Male | E | 11900 | 8220 | 32000 | 18500 | 13461 | 16.5 | 19.6 | 19.8 | 12.9 | 7.98 | 0.027 | 0.046 | 0.012 | 0.014 | 0.012 |  |
| 21 | Male | E | 803 | 470 | 1290 | 1152 | 693 | 2.54 | 6.9 | 2.06 | 3.34 | 1.14 | 0.068 | 0.403 | 0.043 | 0.080 | 0.044 |  |
| 22 | Male | E | -- | -- | 194 | 354 | 442 |  |  | 0.568 | 2.48 | 2.62 |  |  | 0.055 | 0.131 | 0.098 |  |
| 23 | Male | A | 1980 | 1500 | 4380 | 3600 | 583 | 2.88 | 5.24 | 4.08 | 4.63 | 1.4 | 0.030 | 0.070 | 0.019 | 0.027 | 0.049 |  |
| 24 | Male | A | 136 | 108 | 344 | 612 | 532 | 0.67 |  | 0.437 | 2.42 | 0.74 | 0.111 |  | 0.026 | 0.082 | 0.030 |  |
| 25 | Male | A | 276 | 136 | 285 | 235 | 94 | 2.31 | 1.14 | 1.01 | 1.81 | 0.16 | 0.127 | 0.128 | 0.054 | 0.120 | 0.027 |  |
| 26 | Male | A | 627 | 267 | 962 | 721 | 207 |  | 1.2 |  | 2.38 | 0.3 | 0.000 | 0.090 |  | 0.066 | 0.029 |  |
| 27 | Male | A | 602 | 288 | 737 | 697 | 126 | 1.03 | 1.69 |  | 2.14 | 0.34 | 0.033 | 0.114 |  | 0.057 | 0.052 |  |
| 28 | Male | A | 624 | 293 | 912 | 904 | 374 | 1.26 | 0.889 | 0.404 | 1.56 | 0.86 | 0.047 | 0.071 | 0.010 | 0.037 | 0.054 |  |
| 29 | Male | A | 5.5 | 3.9 | 7.8 | 11.6 | 2.5 | 0.0447 |  |  |  |  | 0.161 |  |  |  |  |  |
| 30 | Male | A | 494 | 273 | 592 | 601 | 189 | 1.31 | 4 | 1.66 | 1.89 | 0.32 | 0.052 | 0.289 | 0.060 | 0.063 | 0.033 |  |
| 31 | Male | A | 74.9 | 51.2 | -- | 120 | 76.7 | 0.688 | 0.515 |  | 1.39 |  | 0.214 | 0.235 |  | 0.270 |  |  |
| 32 | Male | A | 543 | -- | 994 | 868 | 503 | 2.24 |  | 1.9 | 2.78 | 4.3 | 0.096 |  | 0.049 | 0.075 | 0.199 |  |
| 33 | Male | A | 16.0 | 6.3 | 23.4 | -- | -- |  |  | 0.44 |  |  |  |  | 0.351 |  |  |  |
| 34 | Male | S | 884 | 583 | 2130 | 1580 | 609 | 2.26 | 2.24 | 9.63 | 3.95 | 2.82 | 0.055 | 0.096 | 0.102 | 0.060 | 0.105 |  |
| 35 | Male | S | 949 | 525 | 1600 | 1305 | 1003 | 2 | 5.25 | 11.7 | 6.58 | 1.9 | 0.042 | 0.264 | 0.135 | 0.101 | 0.038 |  |
| 36 | Male | S | 265 | 172 | 637 | 531 | 603 | 1.47 | 1.05 | 6.17 | 2.42 | 2.36 | 0.106 | 0.117 | 0.181 | 0.085 | 0.069 |  |
| 37 | Male | S | 764 | 318 | 997 | 932 | 608 | 0.668 | 0.776 | 3.33 | 5.24 | 1.27 | 0.021 | 0.057 | 0.078 | 0.131 | 0.049 |  |
| 38 | Male | S | 412 | 245 | 838 | 2100 | 2597 | 2.77 | 0.537 | 5.49 | 16.3 | 26.24 | 0.157 | 0.051 | 0.153 | 0.181 | 0.236 |  |
| 39 | Male | S | 2040 | 988 | 3350 | 2050 | 2011 |  | 12.4 | 0.843 | 9.99 | 13 |  | 0.262 | 0.005 | 0.107 | 0.139 |  |
| 40 | Male | S | -- | 467 | 1190 | 3585 | 1699 |  | 4.34 | 1.46 | 42.3 | 3.72 |  | 0.197 | 0.026 | 0.258 | 0.045 |  |
| 41 | Male | S | 419 | -- | 991 | 1970 | 982 | 0.709 |  | 0.463 | 12.3 | 1.58 | 0.043 |  | 0.012 | 0.151 | 0.040 |  |
| 42 | Male | S | 1890 | -- | -- | 134 | 1028 |  |  |  | 4.18 | 3.82 |  |  |  | 0.674 | 0.084 |  |
| 43 | Male | S | -- | -- | 227 | 241 | 353 |  |  | 6 | 3.64 | 3.14 |  |  | 0.500 | 0.290 | 0.170 |  |
| 44 | Male | R | 142 | 273 | 216 | 173 | 64 | 0.448 | 1.12 | 1.1 | 2.78 | 0.18 | 0.058 | 0.077 | 0.089 | 0.300 | 0.049 |  |
| 45 | Male | R | 2410 | 831 | 2920 | 2350 | 1059 | 4.18 | 15.3 | 5.15 | 7.27 | 4.36 | 0.034 | 0.363 | 0.035 | 0.058 | 0.079 |  |
| 46 | Male | R | -- | -- | 16.6 | 73.6 | 63.7 |  |  | 0.0311 | 0.86 | 0.2 |  |  |  | 0.201 | 0.063 |  |

a: Work assignment, E, A, R, S here represent the electrolytic process department, management office, research and development department, and sulfonation department, respectively.

b: Data not available; the worker did not participate in sampling that year.

**Table S5** Serum and urine PFOS concentrations, renal clearance rate in 46 workers who participated in our investigation at least three times during sampling period.

| No. | Gender | Department | Serum (ng/mL) | | | | | Urine (ng/mL) | | | | | Renal clearance rate(mL/day/kg) | | | | |
| --- | --- | --- | --- | --- | --- | --- | --- | --- | --- | --- | --- | --- | --- | --- | --- | --- | --- |
|  |  |  | 2008 | 2009 | 2010 | 2011 | 2012 | 2008 | 2009 | 2010 | 2011 | 2012 | 2008 | 2009 | 2010 | 2011 | 2012 |
| 1 | Female | E^a^ | 3800 | 2190 | 1910 | 1600 | 1492 |  |  | 1.24 | 0.54 | 4.54 |  |  | 0.017 | 0.009 | 0.085 |
| 2 | Female | E | 1180 | 700 | 2590 | 1485 | 426 | 26.9 |  | 0.416 | 0.25 | 0.28 | 0.544 |  | 0.008 | 0.007 | 0.003 |
| 3 | Female | E | 1060 | 525 | 554 | 401 | 504 |  |  | 0.864 |  | 0.86 | 0.000 |  | 0.025 |  | 0.034 |
| 4 | Female | E | 1590 | 872 | 917 | 883 | 2261 | 0.877 | 0.57 | 0.395 | 1.73 | 0.4 | 0.011 | 0.013 | 0.009 | 0.039 | 0.004 |
| 5 | Female | E | 1450 | 1940 | 2930 | 3170 | 3482 | 4.86 | 0.314 | 1.36 | 0.21 | 1.26 | 0.072 | 0.003 | 0.010 | 0.001 | 0.009 |
| 6 | Female | E | 1280 | 661 | 852 | 563 | -- | 16.7 | 0.504 | 0.971 | 1.07 |  | 0.295 | 0.017 | 0.025 | 0.041 |  |
| 7 | Female | E | 898 | --^b^ | 531 | 382 | 819 | 0.918 |  | 1.14 | 0.14 | 0.7 | 0.018 |  | 0.038 | 0.007 | 0.018 |
| 8 | Female | A | 853 | 478 | 492 | 443 | 750 | 7.8 |  | 0.258 | 0.17 |  | 0.211 |  | 0.013 | 0.009 |  |
| 9 | Female | A | 83 | 64.6 | 162 | -- | -- |  |  |  |  |  |  | 0.028 | 0.000 |  |  |
| 10 | Female | A | 463 | 542 | 875 |  | 2556 | 7.66 |  |  |  |  | 0.361 |  |  |  |  |
| 11 | Female | A | 635 | -- | 339 | 533 | 806 | 0.697 |  | 0.177 | 0.47 |  | 0.031 |  | 0.014 | 0.024 |  |
| 12 | Female | A | 357 | 143 | 176 | -- | -- | 0.425 | 0.361 |  |  |  | 0.031 | 0.066 | 0.000 |  |  |
| 13 | Female | S | 25000 | 16400 | 22900 | 19700 | 19564 | 17.2 | 7.85 | 64.2 | 49.90 | 26.46 | 0.014 | 0.010 | 0.058 | 0.052 | 0.031 |
| 14 | Female | S | 4570 | 3410 | 4060 | 2430 | 2787 |  | 2.17 | 0.496 | 2.18 | 0.46 | 0.000 | 0.013 | 0.002 | 0.018 | 0.004 |
| 15 | Female | S | 4540 | 3700 | 5020 | 4860 | 6775 | 3.94 | 1.4 | 0.884 | 3.31 | 1.62 | 0.021 | 0.009 | 0.004 | 0.016 | 0.005 |
| 16 | Female | R | 460 | 286 | 328 | 350 | 822 | 1.09 |  | 0.714 | 0.43 | 0.22 | 0.055 |  | 0.048 | 0.028 | 0.007 |
| 17 | Female | R | 1130 | 526 | 694 | 600 | 970 |  | 0.831 | 1.14 | 0.54 | 0.44 | 0.000 | 0.037 | 0.039 | 0.021 | 0.013 |
| 18 | Male | E | 3220 | 2090 | 3040 | 2511 | 1890 | 2.12 | 1.51 | 0.912 | 1.32 | 0.58 | 0.017 | 0.018 | 0.008 | 0.017 | 0.005 |
| 19 | Male | E | 894 | 654 | 988 | 4150 | 8501 | 0.736 |  |  | 5.08 | 1.44 | 0.021 |  | 0.000 | 0.029 | 0.004 |
| 20 | Male | E | 4170 | 2790 | 3370 | 2260 | 2761 | 0.943 | 0.198 | 0.855 | 0.47 | 0.18 | 0.004 | 0.001 | 0.005 | 0.004 | 0.001 |
| 21 | Male | E | 3160 | 2120 | 2360 | 2240 | 4028 | 2.18 | 2.73 | 2.44 | 0.52 | 0.5 | 0.015 | 0.035 | 0.028 | 0.006 | 0.003 |
| 22 | Male | E | -- | -- | 799 | 1194 | 3094 |  |  | 0.39 | 1.02 | 0.78 |  |  | 0.009 | 0.016 | 0.004 |
| 23 | Male | A | 2690 | 2110 | 1920 | 1890 | 3890 | 0.818 | 0.47 | 3.98 | 0.29 | 0.1 | 0.006 | 0.004 | 0.041 | 0.003 | 0.001 |
| 24 | Male | A | 650 | 657 | 941 | 1114 | 1744 | 0.217 |  | 0.314 |  | 1.64 | 0.008 |  | 0.007 |  | 0.020 |
| 25 | Male | A | 672 | 424 | 444 | 467 | 613 | 1.09 | 0.288 | 0.777 | 0.75 | 0.1 | 0.025 | 0.010 | 0.027 | 0.025 | 0.003 |
| 26 | Male | A | 620 | 302 | 407 | 411 | 538 |  | 0.078 |  |  | 0.06 | 0.000 | 0.005 |  |  | 0.002 |
| 27 | Male | A | 1220 | 667 | 700 | 556 | 541 | 0.819 | 0.206 |  | 0.51 | 0.26 | 0.013 | 0.006 |  | 0.017 | 0.009 |
| 28 | Male | A | 1150 | 663 | 1010 | 1016 | 1705 | 7.92 | 0.094 | 0.409 | 0.04 | 0.12 | 0.161 | 0.003 | 0.009 | 0.001 | 0.002 |
| 29 | Male | A | 250 | 178 | 165 | 145 | 152 | 0.291 | 0.171 |  |  |  | 0.023 | 0.019 |  |  |  |
| 30 | Male | A | 1110 | 736 | 796 | 1090 | 1474 | 4.7 | 0.84 |  | 0.70 | 0.42 | 0.083 | 0.023 |  | 0.013 | 0.006 |
| 31 | Male | A | 3580 | 3200 | -- | 2450 | 5178 | 7.69 | 1.33 |  | 0.74 |  | 0.050 | 0.010 |  | 0.007 |  |
| 32 | Male | A | 3030 | -- | 1970 | 1778 | 3320 | 3.07 |  | 1.98 | 1.78 | 7.26 | 0.024 |  | 0.026 | 0.023 | 0.051 |
| 33 | Male | A | 1770 | 413 | 479 | -- | -- | 0.465 | 0.14 | 0.139 |  |  | 0.005 | 0.007 | 0.005 |  |  |
| 34 | Male | S | 69100 | 49400 | 50900 | 39700 | 46675 | 22.5 | 1.86 | 14 | 14.70 | 7.8 | 0.007 | 0.001 | 0.006 | 0.009 | 0.004 |
| 35 | Male | S | 5220 | 4030 | 7760 | 8600 | 30601 | 3.09 | 2.58 | 14.2 | 7.74 | 9.72 | 0.012 | 0.017 | 0.034 | 0.018 | 0.006 |
| 36 | Male | S | 1970 | 1450 | 4880 | 4140 | 4208 | 1.92 | 1.8 | 7.14 | 4.91 | 5.64 | 0.019 | 0.024 | 0.027 | 0.022 | 0.023 |
| 37 | Male | S | 3940 | 526 | 2380 | 2580 | 3478 | 1.42 | 0.683 | 3.93 | 2.24 | 1.37 | 0.009 | 0.030 | 0.039 | 0.020 | 0.009 |
| 38 | Male | S | 1850 | 1570 | 1680 | 1986 | 4273 | 2.15 | 0.951 | 6 | 2.80 | 1.18 | 0.027 | 0.014 | 0.083 | 0.033 | 0.006 |
| 39 | Male | S | 2600 | 1680 | 3890 | 4050 | 9011 |  | 0.84 | 1.02 | 0.80 | 6.76 | 0.000 | 0.010 | 0.006 | 0.004 | 0.016 |
| 40 | Male | S | -- | 6150 | 5920 | 6850 | 11552 |  | 0.498 |  | 1.81 | 0.78 |  | 0.002 | 0.000 | 0.006 | 0.001 |
| 41 | Male | S | 3030 | -- | 2100 | 1890 | 4784 | 0.598 |  | 0.418 | 0.48 | 1.1 | 0.005 |  | 0.005 | 0.006 | 0.006 |
| 42 | Male | S | 8430 | -- | -- | 5550 | 16970 |  |  |  | 0.64 | 0.98 | 0.000 |  |  | 0.002 | 0.001 |
| 43 | Male | S | -- | -- | 6490 | 4235 | 7135 |  |  | 6.9 | 5.37 | 1.02 |  |  | 0.020 | 0.024 | 0.003 |
| 44 | Male | R | 1240 | 736 | 765 | 705 | 1043 | 1.07 | 1.27 | 1.17 | 3.45 | 0.38 | 0.016 | 0.032 | 0.027 | 0.091 | 0.006 |
| 45 | Male | R | 7450 | 2380 | 3420 | 3380 | 7305 | 1.56 | 2.77 | 6.54 | 1.59 | 0.22 | 0.004 | 0.023 | 0.038 | 0.009 | 0.001 |
| 46 | Male | R | -- | -- | 142 | 288 | 1158 |  |  |  |  |  |  |  | 0.017 | 0.009 | 0.085 |

a: Work assignment, E, A, R, S here represent the electrolytic process department, management office, research and development department, and sulfonation department, respectively.

b: Data not available; the worker did not participate in sampling that year.

**Table S6** Relationship between the serum PFAA levels and length of service in individual department

| Work assignments | Length of Service | PFHxS | PFOA | PFOS |
| --- | --- | --- | --- | --- |
|  | < 1 year | 8.5 | 9.9 | 140 |
| Management Office | 1-3 years | 65.1 | 48.3 | 641 |
|  | >3 years | 186 | 167 | 728 |
|  | < 1 year | 197 | 118 | 772 |
| Electrolytic Department | 1-3 years | 960 | 918 | 1827 |
|  | >3 years | 1116 | 1160 | 1393 |
|  | < 1 year | 26 | 22 | 318 |
| Research Building | 1-3 years | 946 | 124 | 888 |
|  | >3 years | 607 | 276 | 890 |
|  | < 1 year | 1129 | 185 | 5174 |
| Sulfonation Department | 1-3 years | 1477 | 371 | 5463 |
|  | >3 years | 3087 | 896 | 7756 |

**Table S7** Descriptive statistics for bio-clinical parameters in serum.

| Parameter | normal ranges | N | Detected ranges | Average ± SD | N > upper limit | N < lower limit |
| --- | --- | --- | --- | --- | --- | --- |
| ALT^a^ | (0 - 40) U/L | 248 | 3 - 162 | 27 ± 18 | 37 | -- |
| AST^b^ | (0 - 40) U/L | 248 | 15 - 104 | 28 ± 9 | 23 | -- |
| GGT^c^ | (0 - 50) U/L | 192 | 7 - 233 | 34 ± 33 | 30 | -- |
| BUN^d^ | (1.8 - 8.3) mmol/L | 248 | 1.9 - 11.3 | 5.4 ± 1.5 | 10 | 0 |
| CRE^e^ | (32 - 133) µmol/L | 248 | 44 - 154 | 78 ± 15 | 1 | 0 |
| GLU^f^ | (3.5 - 6.4) mmol/L | 248 | 2.1 - 8.0 | 5.2 ± 0.6 | 9 | 2 |
| CHOL^g^ | (3.12 - 5.69) mmol/L | 248 | 1.3 - 7.2 | 4.8 ± 0.8 | 35 | 5 |
| TG^h^ | (0.48 - 1.71) mmol/L | 248 | 0.22 - 9.31 | 1.41 ± 1.20 | 62 | 19 |
| HDLCh^i^ | (0.78 - 2.0) mmol/L | 248 | 0.8 - 15.0 | 1.5 ± 0.9 | 9 | 0 |
| LDLCh^j^ | (2.07 - 3.40) mmol/L | 248 | 1.13 - 5.07 | 2.81 ± 0.69 | 47 | 28 |
| A1'APOA1^k^ | (1.2 - 1.76) g/L | 248 | 0.96 - 2.20 | 1.41 ± 0.19 | 13 | 25 |
| B'APOB^l^ | (0.66 - 1.07) g/L | 246 | 0.49 - 1.83 | 0.98 ± 0.22 | 67 | 11 |
| a'Lpa^m^ | (0 - 300) mg/L | 247 | 0 - 926 | 126 ± 148 | 25 | -- |
| CHE^n^ | (4300 - 12900) U/L | 133 | 3772 - 16866 | 11027 ± 2232 | 28 | 1 |
| UA^o^ | (89 - 416) µmol/L | 115 | 117 - 504 | 293 ± 81 | 5 | 0 |

a: alanine transaminase; b: aspartate transaminase; c: gamma-glutamyl transferase; d: blood urea nitrogen; e: creatinine; f: blood glucose; g: total cholesterol; h: triglycerides; i: high-density lipoprotein cholesterol; j: low-density lipoprotein cholesterol; k: apolipoprotein A-1; l: [apolipoprotein B](http://www.baidu.com/link?url=XKzdmwgfochq-kl7isSwJtDG_exbAnsCP3fxCnj2v6RmpsgUkQYUBDvtdrcezWjtyIb3KQ1d6Qb_heeTWX6zGD9nSwybEiI0MDzlftF7GRWOM2TORXNkSeNmoqKzW1NQ); m: lipoprotein(a); n: cholinesterase; o: uric acid.
